# Supplementary material for: Uniparental Inheritance Promotes Adaptive Evolution in Cytoplasmic Genomes
Source: Mol Biol Evol. 2016 Dec 25;34(3):677–91. doi: 10.1093/molbev/msw266 (PMC5896580; doi:10.1093/molbev/msw266)
Supplement: Supplementary Data [file msw266_supp.pdf]

Supplementary Material

S1 Supplementary figures

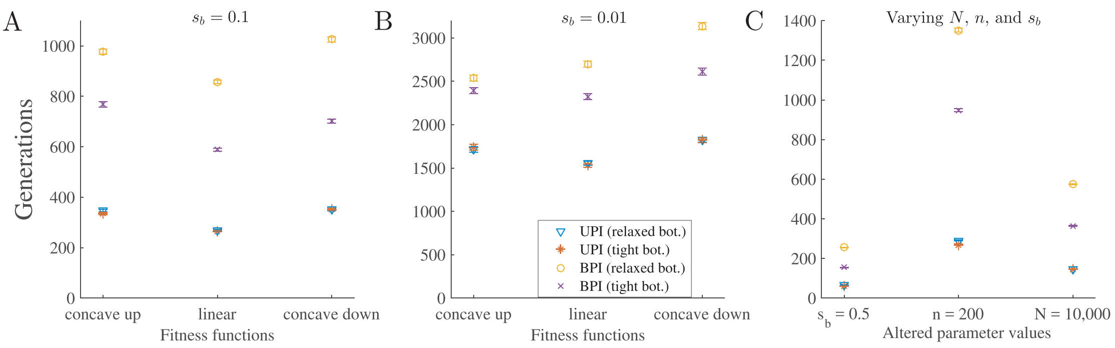

Figure S1: **Time to accumulate a beneficial substitution.** Each plot shows the number of generations to accumulate a beneficial substitution (number of generations before each cytoplasmic genome carries at least $\gamma = 5$ substitutions divided by the mean substitutions per genome in that generation). Parameter values for **A–B**: $N = 1000$, $n = 50$, $\mu_b = 10^{-8}$, and $b = 25$ (relaxed transmission bottleneck) or $b = 5$ (tight transmission bottleneck). **A**. Selection coefficient of 0.1. **B**. Selection coefficient of 0.01. Parameter values for **C** (unless otherwise stated on the x-axis): $N = 1000$, $n = 50$, $\mu_b = 10^{-8}$, $s_b = 0.1$, a linear fitness function for beneficial substitutions, and $b = n/2$ (relaxed transmission bottleneck) or $b = n/10$ (tight transmission bottleneck). Error bars are standard error of the mean.

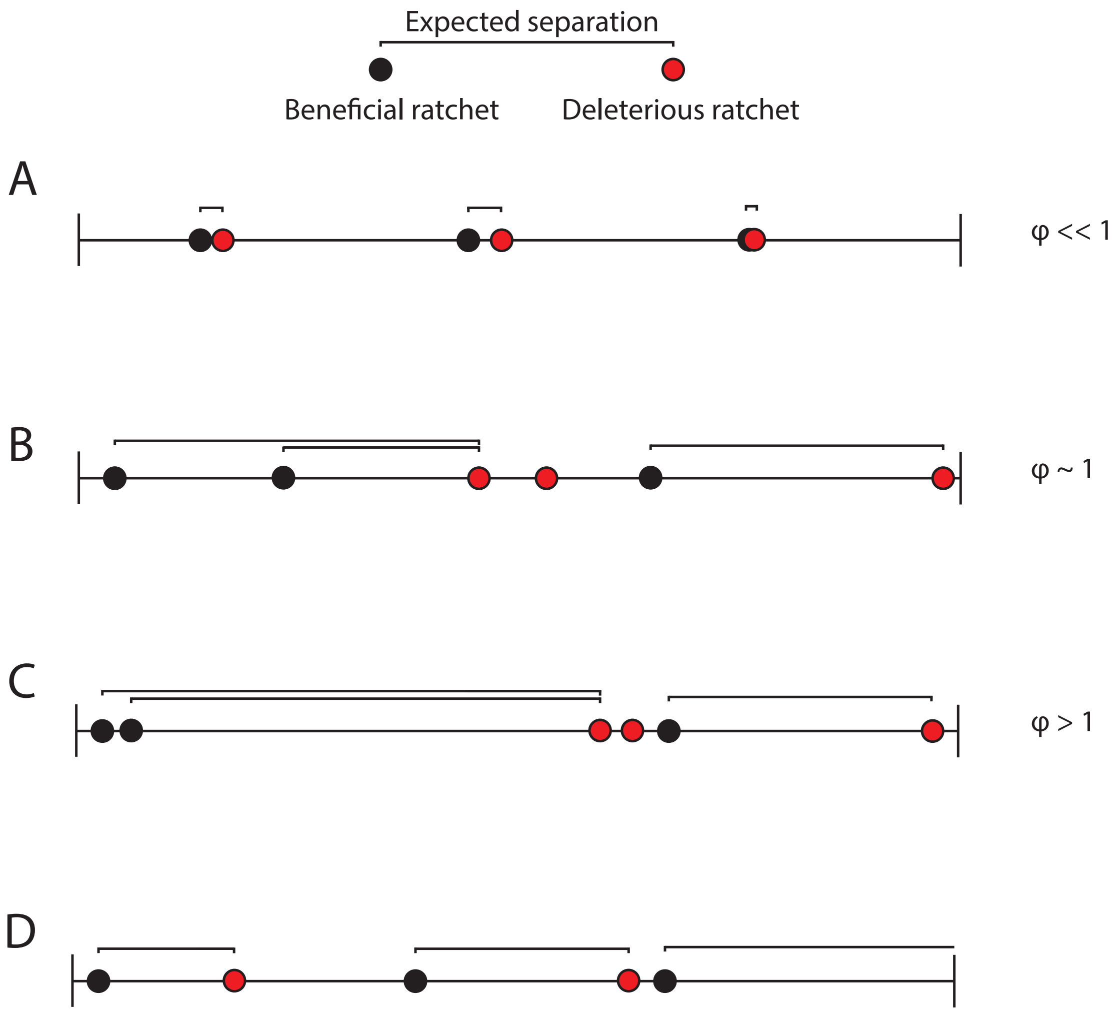
Expected separation
Beneficial ratchet
Deleterious ratchet
A
φ << 1
B
φ ~ 1
C
φ > 1
D

Figure S2 *(previous page)*: **Genetic hitchhiking index**. To calculate the genetic hitchhiking index (ϕ), we compare the number of generations separating beneficial and deleterious ratchets to the number of generations we expect if the two events are uncorrelated. We examine all beneficial ratchets except those involving genomes with > 5 beneficial substitutions (to maintain consistency between the different fitness functions). We map each beneficial ratchet to a single deleterious ratchet but do not limit the number of times a single deleterious ratchet can be mapped to (e.g. **B** and **C**). The expected separation between beneficial and deleterious ratchets for this hypothetical example is shown at the top of the figure. See below for details of how the index is calculated. **A**. When beneficial ratchets are closely followed by deleterious ratchets, $\phi < 1$ and we infer that genetic hitchhiking has occurred. **B**. When the mean of the number of generations separating beneficial and deleterious ratchets are as expected, $\phi \approx 1$ and we infer that the beneficial ratchet does not affect the deleterious ratchet. **C**. When deleterious ratchets follow beneficial ratchets later than expected, $\phi > 1$ and we infer that genetic hitchhiking is suppressed. **D**. When a beneficial ratchet is followed by a deleterious ratchet, we call it a "paired" ratchet. In some instances, the simulation terminates before a deleterious ratchet can follow a beneficial ratchet (an "unpaired" ratchet; e.g. the last beneficial ratchet in **D**). For unpaired ratchets, we add the number of generations separating the beneficial ratchet and the end of the simulation. To calculate the mean generations separating the ratchets, however, we only divide by the number of paired ratchets. Thus, the equation for the index is $\phi = \left[\left(\sum_{i=1}^{n_p}(g_d(i) - g_b(i)) + \sum_{j=1}^{n_u}(g_t - g_b(j))\right)/n_p\right]/\mathbf{E}[s]$. n_p is the total number of paired ratchets, $g_d(i)$ is the generation in which the ith paired deleterious ratchet occurred, and $g_b(i)$ is the generation in which the ith paired beneficial ratchet occurred. n_u is the total number of unpaired ratchets, g_t is the number of generations in each run (10000), and $g_b(j)$ is the generation in which the jth unpaired beneficial ratchet occurred. $\mathbf{E}[s]$ is the expected separation in generations and given by $\mathbf{E}[s] = \left[\left(\sum_{k=1}^{r} g_d(k)/d(k)\right)/r\right] - 1$, where $d(k)$ is the number of deleterious ratchets we considered in the kth simulation, $g_d(k)$ is the generation at which the $d(k)$th deleterious ratchet occurred in the kth simulation, and r is the number of runs for each set of parameter values (500). We subtract 1 because the deleterious ratchets can occur in the same generation as the beneficial ratchet.

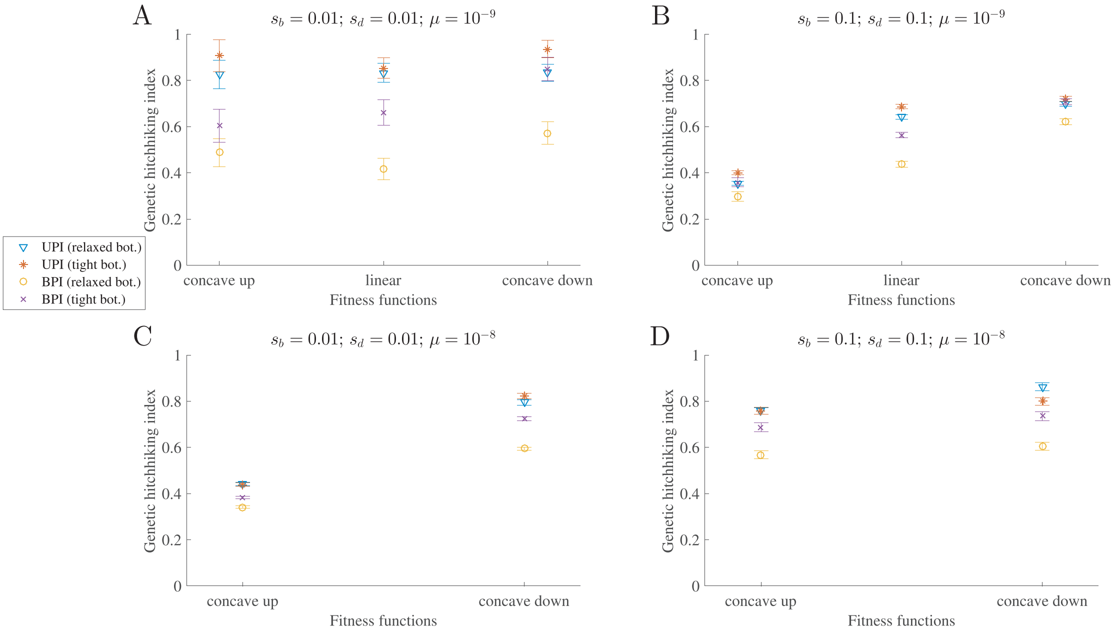

Figure S3: **Genetic hitchhiking when beneficial mutations are rare.** Parameters: $N = 1000$, $n = 50$, $\mu_d = 10^{-7}$, and $b = 25$ (relaxed transmission bottleneck) or $b = 5$ (tight transmission bottleneck). **A** shows $s_b = 0.01$, $s_d = 0.01$, and $\mu_b = 10^{-9}$ while **B** shows $s_b = 0.1$, $s_d = 0.1$, and $\mu_b = 10^{-9}$. **C** shows $s_b = 0.01$, $s_d = 0.01$, and $\mu_b = 10^{-8}$ while **D** shows $s_b = 0.1$, $s_d = 0.1$, and $\mu_b = 10^{-8}$. The plots show the overall level of genetic hitchhiking in each population, measured by our genetic hitchhiking index (see Figure S2 for details). When $\phi < 1$, it indicates the presence of genetic hitchhiking. Error bars are $\pm$ standard error of the mean.

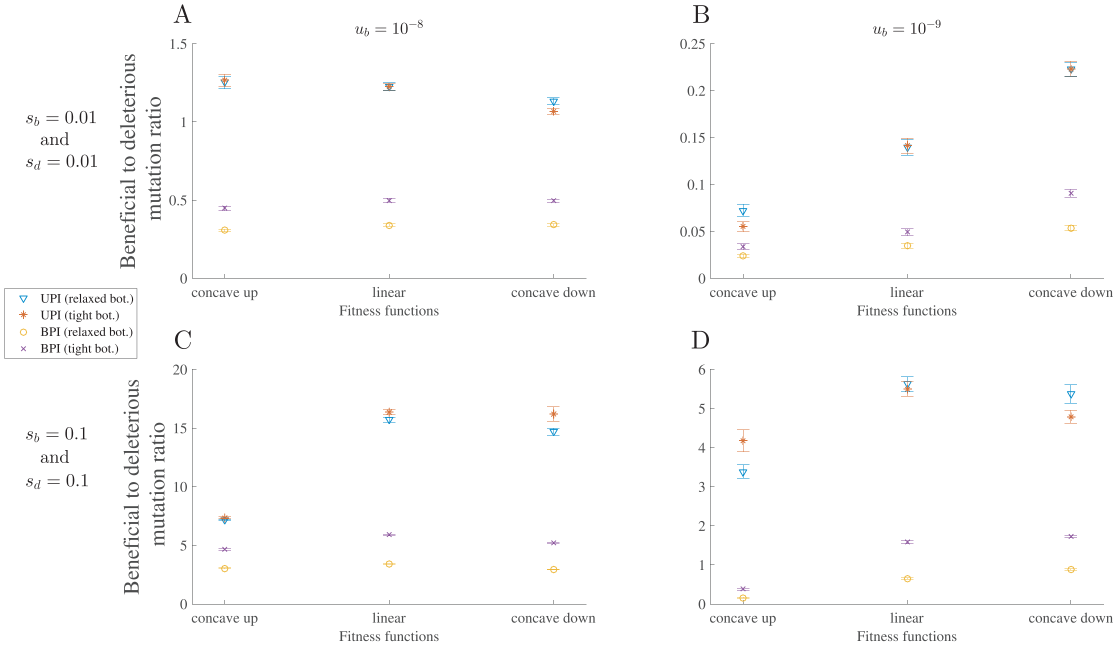

Figure S4: **Ratio of beneficial to deleterious substitutions accumulated under the two inheritance modes.** Parameters: $N = 1000$, $n = 50$, $\mu_d = 10^{-7}$, and $b = 25$ (relaxed transmission bottleneck) or $b = 5$ (tight transmission bottleneck). Panels **A** and **B** show selection coefficients of $s_b = 0.01$, $s_d = 0.01$, while panels **C** and **D** show selection coefficients of $s_b = s_d = 0.1$. For panels **A** and **C**, the beneficial mutation rate is $\mu_b = 10^{-8}$, while for panels **B** and **D** the beneficial mutation rate is $\mu_b = 10^{-9}$. In all cases, uniparental inheritance has a higher ratio of beneficial to deleterious substitutions than biparental inheritance. Error bars are ± standard error of the mean.

S2 Supplementary tables

Table S1: Benchmarking the genetic hitchhiking index using randomly simulated data

Parameters				Results
inheritance	b	s_b	fitness	$\phi \pm$ sd
UPI	$b = 25$	0.01	concave up	1.009±0.040
BPI	$b = 25$	0.01	concave up	1.003±0.040
UPI	$b = 5$	0.01	concave up	0.997±0.047
BPI	$b = 5$	0.01	concave up	1.002±0.040
UPI	$b = 25$	0.01	linear	1.000±0.038
BPI	$b = 25$	0.01	linear	1.005±0.033
UPI	$b = 5$	0.01	linear	0.999±0.039
BPI	$b = 5$	0.01	linear	0.997±0.044
UPI	$b = 25$	0.01	concave down	1.002±0.034
BPI	$b = 25$	0.01	concave down	1.000±0.040
UPI	$b = 5$	0.01	concave down	1.001±0.041
BPI	$b = 5$	0.01	concave down	1.001±0.049
UPI	$b = 25$	0.1	concave up	0.996±0.043
BPI	$b = 25$	0.1	concave up	1.002±0.042
UPI	$b = 5$	0.1	concave up	0.995±0.039
BPI	$b = 5$	0.1	concave up	1.000±0.041
UPI	$b = 25$	0.1	linear	0.995±0.040
BPI	$b = 25$	0.1	linear	0.995±0.038
UPI	$b = 5$	0.1	linear	1.004±0.044
BPI	$b = 5$	0.1	linear	1.000±0.042
UPI	$b = 25$	0.1	concave down	0.996±0.045
BPI	$b = 25$	0.1	concave down	1.001±0.044
UPI	$b = 5$	0.1	concave down	0.998±0.046
BPI	$b = 5$	0.1	concave down	1.004±0.052

Parameters: $N = 1000$, $n = 50$. $\phi \pm$ sd shows the genetic hitchhiking index for randomly simulated datasets ± standard deviation. For each set of parameter values, we determined the expected distance between beneficial and deleterious ratchets. (The expected distance separating beneficial ratchets is $\mathbf{E}[d_b] = \left(\sum_{i=1}^{r} g_b(i)/n_b(i)\right)/r$, where $n_b(i)$ is the number of beneficial ratchets we considered in the ith simulation, $g_b(i)$ is the generation at which the $n_b(i)$th beneficial ratchet occurred in the ith simulation, and r is the

number of runs for each set of parameter values (500). The expected distance separating deleterious ratchets is $\mathbf{E}[d_d] = \left(\sum_{i=1}^{r} g_d(i)/n_d(i)\right)/r$, where $n_d(i)$ is the number of deleterious ratchets we considered in the ith simulation, $g_d(i)$ is the generation at which the $n_d(i)$th deleterious ratchet occurred in the ith simulation, and r is the number of runs for each set of parameter values.) We used these expected values to generate 500 randomly simulated runs, and for each one, used binomial sampling to generate a random number of beneficial and deleterious ratchets. (The number of beneficial ratchets is given by the random variable R_b^i and the number of deleterious ratchets by the random variable R_d^i, where i is the number of the simulated run (out of 500). To obtain R_b^i and R_d^i, we used the R function `rbinom` with parameters $n = 1$, size $= 10000$, and prob $= 1/\mathbf{E}[d_b]$ for beneficial ratchets or prob $= 1/\mathbf{E}[d_d]$ for deleterious ratchets.) For each run, we uniformly sampled R_b^i beneficial and R_d^i deleterious ratchets over 10,000 generations to get the locations of our random beneficial and deleterious ratchets. We then calculated ϕ in the same way as our model-generated data (Figure S2). For each set of parameter values, we repeated this process 100 times, giving us 100 estimates of ϕ. The fifth column shows the mean and standard deviation of these 100 estimates. As can be seen, when beneficial and deleterious ratchets are uncorrelated, $\phi \approx 1$.

S3 Beneficial mutation model

The model is an individual-based model, in which we track all cells in the population (and their gametes). It is written in R version 3.1.2 Team (2013). For each set of parameter values, we ran 500 Monte Carlo simulations. These Monte Carlo simulations were run using packages that enable R code to be run in parallel (`doMC` and `foreach` (Analytics, 2014; Analytics and Weston, 2014)) and produce reproducible output `doRNG` (Gaujoux, 2014)). We ran our simulations on High Performance Computing clusters at The University of Sydney ("Artemis") and National Computational Infrastructure, Australia ("Raijin").

We store the population of cells in a matrix called $\boldsymbol{C}_G^{t,\tau_\zeta}$ that has N rows (each representing an individual cell) and n columns (each representing a cytoplasmic genome). We will use the terminology $\boldsymbol{C}_G^{t,\tau_\zeta}(i,*)$ to refer to the ith row in $\boldsymbol{C}_G^{t,\tau_\zeta}$ (equivalently the ith cell in the population). G represents the inheritance mode and takes values in $\{U, B\}$, where U denotes a cell with uniparental inheritance and B denotes a cell with biparental inheritance. The generation is given by t, while the stage of the life cycle is given by τ_ζ. Thus,

$$\boldsymbol{C}_G^{t,\tau_\zeta} = \begin{bmatrix} \boldsymbol{C}_G^{t,\tau_\zeta}(1,1) & \boldsymbol{C}_G^{t,\tau_\zeta}(1,2) & \dots & \boldsymbol{C}_G^{t,\tau_\zeta}(1,n) \\ \boldsymbol{C}_G^{t,\tau_\zeta}(2,1) & \boldsymbol{C}_G^{t,\tau_\zeta}(2,2) & \dots & \boldsymbol{C}_G^{t,\tau_\zeta}(2,n) \\ \vdots & \vdots & \ddots & \vdots \\ \boldsymbol{C}_G^{t,\tau_\zeta}(N,1) & \boldsymbol{C}_G^{t,\tau_\zeta}(N,2) & \dots & \boldsymbol{C}_G^{t,\tau_\zeta}(N,n) \end{bmatrix},$$

where $\boldsymbol{C}_G^{t,\tau_\zeta}(i,j) = \alpha$ represents α beneficial substitutions in the jth cytoplasmic genome of individual i. Cytoplasmic genomes have l bases, each of which can mutate from a neutral site to a beneficial site. Initially, all genomes have $\alpha = 0$ beneficial substitutions. The first stage of the life cycle is mutation.

S3.1 Mutation

We only consider forward mutation (i.e. genomes can gain beneficial mutations but cannot lose beneficial mutations). We assume that the jth cytoplasmic genome in the ith cell receives $m_{ij}^{b,t}$ new beneficial mutations in generation t, where $m_{ij}^{b,t}$ takes values in $\{0,1,2,3,4,5\}$. The probability that a cytoplasmic genome receives 5 mutations in a single generation is equal to the probability that a genome receives 5 or more mutations (when $\mu_b = 10^{-8}$ and $l = 20000$, the probability that a cytoplasmic genome receives more than 5 mutations in a single generation is calculated by R as 0, so this is a very accurate approximation).

The probability that a genome mutates depends on the mutation rate per base per generation (μ_b), on the number of base pairs available to be mutated ($l - \alpha$), and on the number of mutations that occur ($m_{ij}^{b,t}$). To store these probabilities, we generate a matrix, $\boldsymbol{M}$, with $l+1$ rows (α can take values in $\{0, 1...l\}$) and 5 columns. Thus,

$$\boldsymbol{M} = \begin{bmatrix} \boldsymbol{M}(0,0) & \boldsymbol{M}(0,1) & \boldsymbol{M}(0,2) & \boldsymbol{M}(0,3) & \boldsymbol{M}(0,4) \\ \boldsymbol{M}(1,0) & \boldsymbol{M}(1,1) & \boldsymbol{M}(1,2) & \boldsymbol{M}(1,3) & \boldsymbol{M}(1,4) \\ \boldsymbol{M}(2,0) & \boldsymbol{M}(2,1) & \boldsymbol{M}(2,2) & \boldsymbol{M}(2,3) & \boldsymbol{M}(2,4) \\ \vdots & \vdots & \vdots & \vdots & \vdots \\ \boldsymbol{M}(l,0) & \boldsymbol{M}(l,1) & \boldsymbol{M}(l,2) & \boldsymbol{M}(l,3) & \boldsymbol{M}(l,4) \end{bmatrix}.$$

Each generation, we generate a uniformly random number between 0 and 1, $r_{ij}^{b,t}$, which determines the number of mutations gained by the jth cytoplasmic genome in the ith cell in generation t (i.e. $r_{ij}^{b,t}$ is matched to $\boldsymbol{C}_G^{t,\tau_1}(i,j)$). $r_{ij}^{b,t}$ causes $m_{ij}^{b,t}$ mutations in a genome that already carries α substitutions according to

$$m_{ij}^{b,t} = 5 \text{ if } \quad r_{ij}^{b,t} < \boldsymbol{M}(\alpha, 0),$$

$$m_{ij}^{b,t} = 5 - x \text{ if } \quad \boldsymbol{M}(\alpha, x-1) \leq r_{ij}^{b,t} < \boldsymbol{M}(\alpha, x) \text{ for } 1 \leq x \leq 4$$

$$m_{ij}^{b,t} = 0 \text{ if } \quad r_{ij}^{b,t} \geq \boldsymbol{M}(\alpha, 4)$$

The entries of $\boldsymbol{M}$ are given by

$$\boldsymbol{M}(\alpha, 0) = 1 - \sum_{m_{ij}^{b,t}=0}^{4} \binom{l-\alpha}{m_{ij}^{b,t}} \mu_b^{m_{ij}^{b,t}} (1-\mu_b)^{l-\alpha-m_{ij}^{b,t}}$$

and

$$\boldsymbol{M}(\alpha, x) = 1 - \sum_{m_{ij}^{b,t}=0}^{4} \binom{l-\alpha}{m_{ij}^{b,t}} \mu_b^{m_{ij}^{b,t}} (1-\mu_b)^{l-\alpha-m_{ij}^{b,t}} + \sum_{y=5-x}^{4} \binom{l-\alpha}{y} \mu_b^{y} (1-\mu_b)^{l-\alpha-y} \text{ for } 1 \leq x \leq 4.$$

For the jth cytoplasmic genome in the ith cell, we add the $m_{ij}^{b,t}$ new mutations to the existing α substitutions according to

$$\boldsymbol{C}_G^{t,\tau_2}(i,j) = \boldsymbol{C}_G^{t,\tau_1}(i,j) + m_{ij}^{b,t}$$

S3.2 Selection

The next life cycle stage is selection. Here, each cell is assigned a fitness value based on the number of beneficial cytoplasmic substitutions they carry. The number of beneficial substitutions carried by the ith cell is given by $\beta(i)$, where

$$\beta(i) = \sum_{j=1}^{n} \boldsymbol{C}_G^{t,\tau_2}(i,j).$$

We examine three fitness functions: concave up, linear, and concave down. The fitness of the ith cell under the concave up fitness function is given by

$$\omega_{u,b}\left(\beta\left(i\right)\right) = 1 + s_b \left[\left(\frac{\beta\left(i\right)}{n\gamma}\right)^2 - 1\right],$$

the fitness of the ith cell under the linear fitness function by

$$\omega_{l,b}\left(\beta\left(i\right)\right) = 1 + s_b \left[\frac{\beta\left(i\right)}{n\gamma} - 1\right],$$

and the fitness of the ith cell under the concave down fitness function by

$$\omega_{d,b}\left(\beta\left(i\right)\right) = 1 + s_b \left[\sqrt{\frac{\beta\left(i\right)}{n\gamma}} - 1\right],$$

where γ is the number of beneficial substitutions each cytoplasmic genome must accumulate before the simulation terminates, n is the number of cytoplasmic genomes in each cell, and s_b is the beneficial selection coefficient. We then normalize each cell's fitness so that they sum to 1. The 1-by-N vector $\boldsymbol{S}_G^t$ stores the normalized fitness of the population, where $\boldsymbol{S}_G^t(i)$ gives the relative fitness of the ith cell in the population. To generate $\boldsymbol{S}_G^t$, we first generate a temporary 1-by-N vector, $\boldsymbol{S'}_G^t$ where

$$\boldsymbol{S'}_G^t(i) = \omega_{f,b}\left(\beta(i)\right).$$

where f represents the fitness function used. To generate $\boldsymbol{S}_G^t$, we normalize this vector according to

$$\boldsymbol{S}_G^t(i) = \frac{\boldsymbol{S'}_G^t(i)}{\sum_{z=1}^{N} \boldsymbol{S'}_G^t(z)}.$$

Finally, we feed these probabilities into a multinomial distribution (function `rmultinomial` in the `multinomRob` package (Mebane *et al.*, 2013)) to generate N new cells for the population. Cells can thus die, replace themselves, or produce multiple copies of themselves. We pass the `rmultinomial` function the arguments N and the probability vector $\boldsymbol{S}_G^t$, which generates a 1-by-N vector, $\boldsymbol{O}_G^t$, whose sum is N and whose ith entry represents the number of "offspring" left by the ith cell in the pre-selection population described by $\boldsymbol{C}_G^{t,\tau_2}$. We then use these offspring to reform the post-selection population described by $\boldsymbol{C}_G^{t,\tau_3}$, assuming that each offspring is a perfect copy of its parent. For example, if $\boldsymbol{O}_G^t(i) = 2$ then in $\boldsymbol{C}_G^{t,\tau_3}$ there will be two copies of $\boldsymbol{C}_G^{t,\tau_2}(i,*)$.

S3.3 Gameteogenesis

Each cell produces two gametes: one with mating type A and the other with mating type a.

S3.3.1 Biparental inheritance

To choose which cytoplasmic genomes are passed on, for each mating type we generate a matrix, $\boldsymbol{H}_g^t(i,d) = Y$ with N rows and b columns populated with uniformly random positive integers (Y) in the set $\{1,2,...n\}$, where g represents the nuclear allele of the gamete and when inheritance is biparental takes values in $\{B_A, B_a\}$. $\boldsymbol{H}_g^t(i,d) = Y$ denotes that the dth genome chosen for the new gamete of type g is derived from the Yth cytoplasmic genome of the ith cell. Sampling is with replacement and gametes are stored in a matrix, $\boldsymbol{G}_g^{t,\tau_4}$, which has N rows and b columns. $\boldsymbol{G}_{B_A}^{t,\tau_4}(i,d)$ is produced by

$$\boldsymbol{G}_{B_A}^{t,\tau_4}(i,d) = \boldsymbol{C}_B^{t,\tau_3}(i, \boldsymbol{H}_{B_A}^t(i,d) = Y).$$

$\boldsymbol{G}_{B_a}^{t,\tau_4}(i,d)$ is produced by

$$\boldsymbol{G}_{B_a}^{t,\tau_4}(i,d) = \boldsymbol{C}_B^{t,\tau_3}(i, \boldsymbol{H}_{B_a}^t(i,d) = Y).$$

S3.3.2 Uniparental inheritance

When inheritance is uniparental, g takes values in $\{U_A, U_a\}$. $\boldsymbol{G}_{U_A}^{t,\tau_4}(i,d)$ is produced by

$$\boldsymbol{G}_{U_A}^{t,\tau_4}(i,d) = \boldsymbol{C}_U^{t,\tau_3}(i, \boldsymbol{H}_{U_A}^t(i,d) = Y),$$

and $\boldsymbol{G}_{U_a}^{t,\tau_4}(i,d)$ is produced by

$$\boldsymbol{G}_{B_a}^{t,\tau_4}(i,d) = \boldsymbol{C}_U^{t,\tau_3}(i, \boldsymbol{H}_{U_a}^t(i,d) = Y).$$

S3.4 Random mating

S3.4.1 Biparental inheritance

Biparental inheritance simply combines the cytoplasmic genomes of both gametes. For each of the B_A- and B_a-carrying gametes, we generate a 1-by-N vector, $\boldsymbol{T}_g^t(i) = Z$ that contains a random ordering (without replacement) of positive integers from the set $\{1, 2, ...N\}$. We use these vectors to pair up gametes according to

$$\boldsymbol{C'}_B^{t+1,\tau_1}(i,*) = \boldsymbol{G}_{B_A}^{t,\tau_4}(\boldsymbol{T}_{B_A}^t(i) = Z, *) \| \boldsymbol{G}_{B_a}^{t,\tau_4}(\boldsymbol{T}_{B_a}^t(i) = Z, *),$$

where $\|$ indicates that the two vectors are concatenated. $\boldsymbol{C'}_B^{t+1,\tau_1}$ is a temporary matrix (to be replaced by $\boldsymbol{C}_B^{t+1,\tau_1}$), which contains $2b$ columns (representing the $2b$ genomes). Since $2b < n$ when we impose a transmission bottleneck, the final step for each cell is to sample n genomes with replacement from these $2b$ genomes (we include this step even when the transmission bottleneck is relaxed and $2b = n$). This sampling follows the same approach as described in gameteogenesis, but now instead of choosing b genomes from a cell with n genomes, we choose n genomes from a cell with $2b$ genomes. We generate a matrix, $\boldsymbol{F}_B^t(i,j) = Q$ with N rows and n columns populated with uniformly random positive integers sampled with replacement from the set $\{1, 2, ...2b\}$, which we use to

sample the new genomes according to

$$\boldsymbol{C}_{B}^{t+1,\tau_1}(i,j) = \boldsymbol{C'}_{B}^{t+1,\tau_1}(i, \boldsymbol{F}_{G}^{t}(i,j) = Q).$$

S3.4.2 Uniparental inheritance

Under uniparental inheritance, only the gamete with mating type A passes on its cytoplasmic genomes. Thus, to pair up gametes we only need to generate one 1-by-N vector, $\boldsymbol{T}_{U_A}^{t}(i) = Z$ that contains a random ordering (without replacement) of positive integers in the set $\{1, 2, ...N\}$, giving

$$\boldsymbol{C}_{U}^{t+1,\tau_1}(i,*) = \boldsymbol{G}_{U_A}^{t,\tau_4}(\boldsymbol{T}_{U_A}^{t}(i) = Z, *).$$

(Note, randomly ordering the U_A gametes is not strictly necessary, but we do it to be consistent with the model of biparental inheritance.) Now $\boldsymbol{C}_{U}^{t+1,\tau_1}(i,*)$ only contains b columns (representing b genomes), so for each cell we sample n genomes with replacement from these b genomes. We generate a matrix, $\boldsymbol{F}_{U}^{t}(i,j) = Q$ with N rows and n columns populated with uniformly random positive integers sampled with replacement from the set $\{1, 2, ...b\}$. We use this to sample the new genomes according to

$$\boldsymbol{C}_{U}^{t+1,\tau_1}(i,j) = \boldsymbol{C'}_{U}^{t+1,\tau_1}(i, \boldsymbol{F}_{U}^{t}(i,j) = Q).$$

S4 Deleterious mutation model

This model differs from the previous model in how it deals with selection. Mutations are now deleterious, not beneficial. Each cell is assigned a fitness value based on the number of deleterious cytoplasmic substitutions it carries. The number of deleterious substitutions carried by the ith cell is given by $\rho(i)$, where

$$\rho(i) = \sum_{j=1}^{n} \boldsymbol{C}_G^{t,\tau_2}(i,j).$$

For deleterious mutations, we examine the concave down (decreasing) fitness function. The fitness of the ith cell is given by

$$\omega_{d,d}\left(\rho\left(i\right)\right) = 1 - s_d\left(\frac{\rho\left(i\right)}{n\gamma}\right)^2,$$

where n is the number of cytoplasmic genomes in each cell, and s_d is the deleterious selection coefficient. To maintain consistency with the model that considers only beneficial mutations, γ is set to the same value as in the first model. If $\omega_{d,d}\left(\rho\left(i\right)\right) < 0$ we set $\omega_{d,d}\left(\rho\left(i\right)\right) = 0$ (as fitness cannot be negative). Everything else proceeds as detailed in section S3.2.

S5 Beneficial and deleterious mutation model

In this version of the model, we store the population of cells in a matrix called $\boldsymbol{C}_G^{t,\tau_\zeta}$ that has $2N$ rows and n columns. $\boldsymbol{C}_G^{t,\tau_\zeta}(i,j)$ stores the number of beneficial substitutions in the jth genome of the ith cell, while $\boldsymbol{C}_G^{t,\tau_\zeta}(i+N,j)$ stores the number of deleterious substitutions in the jth genome of the ith cell. As before, G represents the inheritance mode and takes values in $\{U,B\}$. The generation is given by t, while the stage of the life cycle is given by τ_ζ. Thus,

$$\boldsymbol{C}_G^{t,\tau_\zeta} = \begin{bmatrix} \boldsymbol{C}_G^{t,\tau_\zeta}(1,1) & \boldsymbol{C}_G^{t,\tau_\zeta}(1,2) & \dots & \boldsymbol{C}_G^{t,\tau_\zeta}(1,n) \\ \boldsymbol{C}_G^{t,\tau_\zeta}(2,1) & \boldsymbol{C}_G^{t,\tau_\zeta}(2,2) & \dots & \boldsymbol{C}_G^{t,\tau_\zeta}(2,n) \\ \vdots & \vdots & \ddots & \vdots \\ \boldsymbol{C}_G^{t,\tau_\zeta}(2N,1) & \boldsymbol{C}_G^{t,\tau_\zeta}(2N,2) & \dots & \boldsymbol{C}_G^{t,\tau_\zeta}(2N,n) \end{bmatrix},$$

where $\boldsymbol{C}_G^{t,\tau_\zeta}(i,j) = \alpha$ and $\boldsymbol{C}_G^{t,\tau_\zeta}(i+N,j) = \kappa$ represent α beneficial substitutions and κ deleterious substitutions respectively in the jth cytoplasmic genome of individual i. Cytoplasmic genomes have l bases, each of which can change from a neutral site to a beneficial or deleterious substitution. Initially, all genomes have $\alpha = 0$ beneficial substitutions and $\kappa = 0$ deleterious substitutions. The first stage of the life cycle is mutation.

S5.1 Mutation

We assume that the jth cytoplasmic genome in the ith cell gains $m_{ij}^{b,t}$ new beneficial mutations in generation t, and $m_{ij}^{d,t}$ new deleterious mutations in generation t, where both $m_{ij}^{b,t}$ and $m_{ij}^{d,t}$ take values in $\{0,1,2,3,4,5\}$. We store the probabilities of gaining $m_{ij}^{b,t}$ beneficial mutations in a matrix, $\boldsymbol{M_b}$, with $l+1$ rows (representing the possible states that a cytoplasmic genome can take) and 5 columns. Thus,

$$
\boldsymbol{M_b} = \begin{bmatrix} \boldsymbol{M_b}(0,0) & \boldsymbol{M_b}(0,1) & \boldsymbol{M_b}(0,2) & \boldsymbol{M_b}(0,3) & \boldsymbol{M_b}(0,4) \\ \boldsymbol{M_b}(1,0) & \boldsymbol{M_b}(1,1) & \boldsymbol{M_b}(1,2) & \boldsymbol{M_b}(1,3) & \boldsymbol{M_b}(1,4) \\ \boldsymbol{M_b}(2,0) & \boldsymbol{M_b}(2,1) & \boldsymbol{M_b}(2,2) & \boldsymbol{M_b}(2,3) & \boldsymbol{M_b}(2,4) \\ \vdots & \vdots & \vdots & \vdots & \vdots \\ \boldsymbol{M_b}(l,0) & \boldsymbol{M_b}(l,1) & \boldsymbol{M_b}(l,2) & \boldsymbol{M_b}(l,3) & \boldsymbol{M_b}(l,4) \end{bmatrix}.
$$

Likewise, we store the probabilities of gaining $m_{ij}^{d,t}$ deleterious mutations in a matrix, $\boldsymbol{M_d}$, given by

$$
\boldsymbol{M_d} = \begin{bmatrix} \boldsymbol{M_d}(0,0) & \boldsymbol{M_d}(0,1) & \boldsymbol{M_d}(0,2) & \boldsymbol{M_d}(0,3) & \boldsymbol{M_d}(0,4) \\ \boldsymbol{M_d}(1,0) & \boldsymbol{M_d}(1,1) & \boldsymbol{M_d}(1,2) & \boldsymbol{M_d}(1,3) & \boldsymbol{M_d}(1,4) \\ \boldsymbol{M_d}(2,0) & \boldsymbol{M_d}(2,1) & \boldsymbol{M_d}(2,2) & \boldsymbol{M_d}(2,3) & \boldsymbol{M_d}(2,4) \\ \vdots & \vdots & \vdots & \vdots & \vdots \\ \boldsymbol{M_d}(l,0) & \boldsymbol{M_d}(l,1) & \boldsymbol{M_d}(l,2) & \boldsymbol{M_d}(l,3) & \boldsymbol{M_d}(l,4) \end{bmatrix}.
$$

Each generation, we generate two uniformly random numbers between 0 and 1, $r_{ij}^{b,t}$ and $r_{ij}^{d,t}$, where $r_{ij}^{b,t}$ determines the number of beneficial mutations gained by the jth cytoplasmic genome in the ith cell in generation t and $r_{ij}^{d,t}$ determines the number of deleterious mutations gained by the jth cytoplasmic genome in the ith cell in generation t (i.e. $r_{ij}^{b,t}$ is matched to $\boldsymbol{C}_G^{t,\tau_1}(i,j)$ and $r_{ij}^{d,t}$ is matched to $\boldsymbol{C}_G^{t,\tau_1}(N+i,j)$). $r_{ij}^{b,t}$ causes $m_{ij}^{b,t}$ beneficial mutations in the jth genome of the ith cell, which already carries $\alpha+\kappa$ mutations according to

$$
m_{ij}^{b,t} = 5 \text{ if } \quad r_{ij}^{b,t} < \boldsymbol{M_b}(\alpha+\kappa, 0),
$$

$$m_{ij}^{b,t} = 5 - x \text{ if } \quad \boldsymbol{M_b}(\alpha + \kappa, x - 1) \leq r_{ij}^{b,t} < \boldsymbol{M_b}(\alpha + \kappa, x) \text{ for } 1 \leq x \leq 4,$$

$$m_{ij}^{b,t} = 0 \text{ if } \quad r_{ij}^{b,t} \geq \boldsymbol{M_b}(\alpha + \kappa, 4).$$

The entries of $\boldsymbol{M_b}$ are given by

$$\boldsymbol{M_b}(\alpha + \kappa, 0) = 1 - \sum_{m_{ij}^{b,t}=0}^{4} \binom{l - \alpha - \kappa}{m_{ij}^{b,t}} \mu_b^{m_{ij}^{b,t}} (1 - \mu_b)^{l - \alpha - \kappa - m_{ij}^{b,t}}$$

and

$$\begin{aligned}\boldsymbol{M_b}(\alpha + \kappa, x) = 1 - &\sum_{m_{ij}^{b,t}=0}^{4} \binom{l - \alpha - \kappa}{m_{ij}^{b,t}} \mu_b^{m_{ij}^{b,t}} (1 - \mu_b)^{l - \alpha - \kappa - m_{ij}^{b,t}} \\ &+ \sum_{y=5-x}^{4} \binom{l - \alpha - \kappa}{y} \mu_b^{y} (1 - \mu_b)^{l - \alpha - \kappa - y} \text{ for } 1 \leq x \leq 4.\end{aligned}$$

$r_{ij}^{d,t}$ causes $m_{ij}^{d,t}$ deleterious mutations in the jth genome of the ith cell, which already carries $\alpha + \kappa$ mutations according to

$$m_{ij}^{d,t} = 5 \text{ if } \quad r_{ij}^{d,t} < \boldsymbol{M_d}(\alpha+\kappa, 0),$$

$$m_{ij}^{d,t} = 5 - x \text{ if } \quad \boldsymbol{M_d}(\alpha+\kappa, x-1) \leq r_{ij}^{d,t} < \boldsymbol{M_d}(\alpha+\kappa, x) \text{ for } 1 \leq x \leq 4,$$

$$m_{ij}^{d,t} = 0 \text{ if } \quad r_{ij}^{d,t} \geq \boldsymbol{M_d}(\alpha+\kappa, 4).$$

The entries of $\boldsymbol{M_d}$ are given by

$$\boldsymbol{M_d}(\alpha+\kappa, 0) = 1 - \sum_{m_{ij}^{d,t}=0}^{4} \binom{l-\alpha-\kappa}{m_{ij}^{d,t}} \mu_d^{m_{ij}^{d,t}} (1-\mu_d)^{l-\alpha-\kappa-m_{ij}^{d,t}}$$

and

$$\boldsymbol{M_d}(\alpha+\kappa, x) = 1 - \sum_{m_{ij}^{d,t}=0}^{4} \binom{l-\alpha-\kappa}{m_{ij}^{d,t}} \mu_d^{m_{ij}^{d,t}} (1-\mu_d)^{l-\alpha-\kappa-m_{ij}^{d,t}}$$

$$+ \sum_{y=5-x}^{4} \binom{l-\alpha-\kappa}{y} \mu_d^{y} (1-\mu_d)^{l-\alpha-\kappa-y} \text{ for } 1 \leq x \leq 4.$$

For the jth cytoplasmic genome in the ith cell, we add the $m_{ij}^{b,t}$ new beneficial mutations to the existing α beneficial mutations and the $m_{ij}^{d,t}$ new deleterious mutations to the existing κ beneficial mutations according to

$$\boldsymbol{C}_G^{t,\tau_2}(i,j) = \boldsymbol{C}_G^{t,\tau_1}(i,j) + m_{ij}^{b,t},$$

and

$$\boldsymbol{C}_G^{t,\tau_2}(i+N,j) = \boldsymbol{C}_G^{t,\tau_1}(i+N,j) + m_{ij}^{d,t}.$$

S5.2 Selection

The next life cycle stage is selection. Here, each cell is assigned a fitness value based on the number of beneficial and deleterious substitutions they carry. The number of beneficial substitutions carried by the ith cell is given by $\beta(i)$ and the number of deleterious substitutions carried by the ith cell is $\rho(i)$, where

$$\beta(i) = \sum_{j=1}^{n} \boldsymbol{C}_G^{t,\tau_2}(i,j),$$

and

$$\rho(i) = \sum_{j=1}^{n} \boldsymbol{C}_G^{t,\tau_2}(i+N, j).$$

We examine concave down fitness (decreasing) for deleterious substitutions, and concave up, linear, and concave down fitness functions for beneficial substitutions. The fitness of the ith cell, which carries $\beta(i)$ beneficial substitutions and $\rho(i)$ deleterious substitutions under the concave up fitness function for beneficial substitutions is given by

$$\omega_{u,bd}\left(\beta\left(i\right), \rho\left(i\right)\right) = 1 + s_b \left[\left(\frac{\beta\left(i\right)}{n\gamma}\right)^2 - 1\right] - s_d \left(\frac{\rho\left(i\right)}{n\gamma}\right)^2,$$

its fitness under the linear fitness function for beneficial substitutions is given by

$$\omega_{l,bd}\left(\beta\left(i\right), \rho\left(i\right)\right) = 1 + s_b \left(\frac{\beta\left(i\right)}{n\gamma} - 1\right) - s_d \left(\frac{\rho\left(i\right)}{n\gamma}\right)^2,$$

and its fitness under the concave down fitness function for beneficial substitutions is given by

$$\omega_{d,bd}\left(\beta\left(i\right), \rho\left(i\right)\right) = 1 + s_b \left(\sqrt{\frac{\beta\left(i\right)}{n\gamma}} - 1\right) - s_d \left(\frac{\rho\left(i\right)}{n\gamma}\right)^2,$$

where n is the number of cytoplasmic genomes in each cell, s_b is the beneficial selection coefficient and s_d is the deleterious selection coefficient. To maintain consistency with the first two models, γ is set to the same value as in the model with beneficial mutations only. If $\omega_{f,bd}\left(\beta\left(i\right), \rho\left(i\right)\right) < 1$ we set $\omega_{f,bd}\left(\beta\left(i\right), \rho\left(i\right)\right) = 0$ (as fitness cannot be negative).

The 1-by-N vector $\boldsymbol{S}_G^t$ stores the normalized fitness of the population, where $\boldsymbol{S}_G^t(i)$ gives

the relative fitness of the ith cell in the population. To generate $\boldsymbol{S}_G^t$, we first generate a temporary 1-by-N matrix, $\boldsymbol{S'}_G^t$ where $\boldsymbol{S'}_G^t(i) = \omega_{f,bd}\left(\beta(i), \rho(i)\right)$. To generate $\boldsymbol{S}_G^t$, we normalize this vector according to

$$\boldsymbol{S}_G^t(i) = \frac{\boldsymbol{S'}_G^t(i)}{\sum\limits_{z=1}^{N} \boldsymbol{S'}_G^t(z)}.$$

Finally, we use the probabilities in $\boldsymbol{S}_G^t$ to generate N new cells for the population, using the process described in section S3.2.

S5.3 Gameteogenesis

S5.3.1 Biparental inheritance

To choose which cytoplasmic genomes are passed on, for each mating type we generate a matrix, $\boldsymbol{H}_g^t(i,d) = Y$ with N rows and b columns populated with uniformly random positive integers (Y) in the set $\{1, 2, ...n\}$, where g represents the nuclear allele of the gamete and when inheritance is biparental takes values in $\{B_A, B_a\}$. $\boldsymbol{H}_g^t(i,d) = Y$ denotes that the dth genome chosen for the new gamete of type g is derived from the Yth cytoplasmic genome of the ith cell. Sampling is with replacement and gametes are stored in a matrix, $\boldsymbol{G}_g^{t,\tau_4}$ which has $2N$ rows and b columns. Since the beneficial substitutions of the dth genome of the ith gamete is stored in $\boldsymbol{G}_g^{t,\tau_4}(i,d)$ and the deleterious substitutions of the dth genome of the ith gamete are stored in $\boldsymbol{G}_g^{t,\tau_4}(i+N,d)$, both must segregate together. $\boldsymbol{G}_{B_A}^{t,\tau_4}(i,d)$ is produced by

$$\boldsymbol{G}_{B_A}^{t,\tau_4}(i,d) = \boldsymbol{C}_B^{t,\tau_3}(i, \boldsymbol{H}_{B_A}^t(i,d) = Y),$$

and

$$\boldsymbol{G}_{B_A}^{t,\tau_4}(i+N,d) = \boldsymbol{C}_{B}^{t,\tau_3}(i+N, \boldsymbol{H}_{B_A}^{t}(i,d) = Y).$$

$\boldsymbol{G}_{B_a}^{t,\tau_4}(i,d)$ is produced by

$$\boldsymbol{G}_{B_a}^{t,\tau_4}(i,d) = \boldsymbol{C}_{B}^{t,\tau_3}(i, \boldsymbol{H}_{B_a}^{t}(i,d) = Y),$$

and

$$\boldsymbol{G}_{B_a}^{t,\tau_4}(i+N,d) = \boldsymbol{C}_{B}^{t,\tau_3}(i+N, \boldsymbol{H}_{B_a}^{t}(i,d) = Y).$$

S5.3.2 Uniparental inheritance

When inheritance is uniparental, $\boldsymbol{G}_{U_A}^{t,\tau_4}(i,d)$ is produced by

$$\boldsymbol{G}_{U_A}^{t,\tau_4}(i,d) = \boldsymbol{C}_{U}^{t,\tau_3}(i, \boldsymbol{H}_{U_A}^{t}(i,d) = Y),$$

and

$$\boldsymbol{G}_{U_A}^{t,\tau_4}(i+N,d) = \boldsymbol{C}_{U}^{t,\tau_3}(i+N, \boldsymbol{H}_{U_A}^{t}(i,d) = Y).$$

$\boldsymbol{G}_{U_a}^{t,\tau_4}(i,d)$ is produced by

$$\boldsymbol{G}_{U_a}^{t,\tau_4}(i,d) = \boldsymbol{C}_{U}^{t,\tau_3}(i, \boldsymbol{H}_{U_a}^{t}(i,d) = Y),$$

and

$$\boldsymbol{G}_{U_a}^{t,\tau_4}(i+N,d) = \boldsymbol{C}_{U}^{t,\tau_3}(i+N, \boldsymbol{H}_{U_a}^{t}(i,d)=Y).$$

S5.4 Random mating

S5.4.1 Biparental inheritance

Biparental inheritance simply combines the cytoplasmic genomes of both gametes. For each of the B_A- and B_a-carrying gametes, we generate a 1-by-N vector, $\boldsymbol{T}_g^t(i) = Z$ that contains a random ordering (without replacement) of positive integers from the set $\{1, 2, ...N\}$. We use these vectors to pair up gametes according to

$$\boldsymbol{C'}_{B}^{t+1,\tau_1}(i,*) = \boldsymbol{G}_{B_A}^{t,\tau_4}(\boldsymbol{T}_{B_A}^t(i)=Z,*) \| \boldsymbol{G}_{B_a}^{t,\tau_4}(\boldsymbol{T}_{B_a}^t(i)=Z,*),$$

and

$$\boldsymbol{C'}_{B}^{t+1,\tau_1}(i+N,*) = \boldsymbol{G}_{B_A}^{t,\tau_4}\left(\left(\boldsymbol{T}_{B_A}^t(i)=Z\right)+N,*\right) \| \boldsymbol{G}_{B_a}^{t,\tau_4}\left(\left(\boldsymbol{T}_{B_a}^t(i)=Z\right)+N,*\right).$$

$\|$ indicates that the two vectors are concatenated. $\boldsymbol{C'}_{B}^{t+1,\tau_1}$ is a temporary matrix (to be replaced by $\boldsymbol{C}_{B}^{t+1,\tau_1}$), which contains $2b$ columns (representing $2b$ genomes). Since $2b < n$ when we impose a transmission bottleneck, the final step for each cell is to sample n genomes with replacement from these $2b$ genomes. This sampling follows the same approach as described in meiosis, but now instead of choosing b genomes from a cell with n genomes, we choose n genomes from a cell with $2b$ genomes. We generate a matrix, $\boldsymbol{F}_B^t(i,j) = Q$ with N rows and n columns populated with uniformly random positive integers sampled with replacement from the set $\{1, 2, ...2b\}$, which we use to

sample the new genomes according to

$$\boldsymbol{C}_B^{t+1,\tau_1}(i,j) = \boldsymbol{C'}_B^{t+1,\tau_1}(i, \boldsymbol{F}_B^t(i,j) = Q),$$

and

$$\boldsymbol{C}_B^{t+1,\tau_1}(i+N,j) = \boldsymbol{C'}_B^{t+1,\tau_1}(i+N, \boldsymbol{F}_B^t(i,j) = Q).$$

S5.4.2 Uniparental inheritance

Under uniparental inheritance, only the gamete with mating type A passes on its cytoplasmic genomes. Thus, to pair up gametes we only need to generate one 1-by-N vector, $\boldsymbol{T}_{U_A}^t(i) = Z$ that contains a random ordering (without replacement) of positive integers in the set $\{1, 2, ...N\}$, giving

$$\boldsymbol{C}_U^{t+1,\tau_1}(i,*) = \boldsymbol{G}_{U_A}^{t,\tau_4}(\boldsymbol{T}_{U_A}^t(i) = Z, *),$$

and

$$\boldsymbol{C}_U^{t+1,\tau_1}(i+N,*) = \boldsymbol{G}_{U_A}^{t,\tau_4}\left(\left(\boldsymbol{T}_{U_A}^t(i) = Z\right) + N, *\right).$$

Now $\boldsymbol{C}_U^{t+1,\tau_1}(i,*)$ only contains b columns (representing b genomes), so for each cell we sample n genomes with replacement from these b genomes. We generate a matrix, $\boldsymbol{F}_U^t(i,j) = Q$ with N rows and n columns populated with uniformly random positive integers sampled with replacement from the set $\{1, 2, ...b\}$. We use this to sample the new genomes according to

$$\boldsymbol{C}_U^{t+1,\tau_1}(i,j) = \boldsymbol{C'}_U^{t+1,\tau_1}(i, \boldsymbol{F}_U^t(i,j) = Q),$$

and

$$\boldsymbol{C}_U^{t+1,\tau_1}(i+N,j) = \boldsymbol{C'}_U^{t+1,\tau_1}(i+N, \boldsymbol{F}_U^t(i,j) = Q).$$

S6 Free-living genome model

In our model of free-living genomes, we store the population of cells in a 1-by-N_{FL} vector (or 1-by-$2N_{FL}$ vector for the model with both beneficial and deleterious mutations), where $N_{FL} = N \times n$ or $N_{FL} = N$, depending on whether the free-living population size is matched to the number of cytoplasmic genomes or eukaryote hosts. In the model that only considers beneficial mutations, $\boldsymbol{C}^{t,\tau_\varsigma}(i) = \alpha$ indicates that the ith free-living cell carries α substitutions. In the model that only considers deleterious mutations, $\boldsymbol{C}^{t,\tau_\varsigma}(i) = \kappa$ indicates that the ith free-living cell carries κ substitutions. In the model that considers both beneficial and deleterious mutations, $\boldsymbol{C}^{t,\tau_\varsigma}(i) = \alpha$ and $\boldsymbol{C}^{t,\tau_\varsigma}(i + N_{FL}) = \kappa$ indicates that the ith free-living cell carries α beneficial and κ deleterious substitutions.

There are two stages to the free-living life cycle: mutation and selection. Mutation proceeds in the same way as it does in the model of cytoplasmic genomes (but now the uniformly random number r_i^t is matched to the ith cell in the population). Selection now acts directly on free-living genomes rather than on host cells that carry multiple cytoplasmic genomes. To modulate the degree to which free-living mutations affect fitness (relative to cytoplasmic genomes), we include an additional parameter, s_{FL}. For example, the fitness of the ith cell ($\boldsymbol{C}^{t,\tau_\varsigma}(i) = \alpha$) under the linear fitness function in the model that considers beneficial mutations only is

$$\omega_{l,b}\left(\boldsymbol{C}^{t,\tau_\varsigma}(i)\right) = 1 + s_b\left[\frac{\alpha s_{FL}}{n\gamma} - 1\right].$$

Based on these fitness values, we generate a 1-by-N_{FL} normalized fitness vector, which we use to choose N_{FL} cells by multinomial sampling for the new population, as described in section S3.2.

References

Analytics, R. 2014. *doMC: Foreach parallel adaptor for the multicore package*. R package version 1.3.3. [S3]

Analytics, R. and Weston, S. 2014. *foreach: Foreach looping construct for R*. R package version 1.4.2. [S3]

Gaujoux, R. 2014. *doRNG: Generic Reproducible Parallel Backend for foreach Loops*. R package version 1.6. [S3]

Mebane, W. R., Jr., and Sekhon, J. S. 2013. *multinomRob: Robust Estimation of Overdispersed Multinomial Regression Models*. R package version 1.8-6.1. [S3.2]

Team, R. C. 2013. R: A language and environment for statistical computing. [S3]
